# Expression Level and Clinical Significance of NKILA in Human Cancers: A Systematic Review and Meta-Analysis

**DOI:** 10.1155/2020/4540312

**Published:** 2020-08-11

**Authors:** Siyuan Tian, Yang Yu, Honghua Huang, Aibing Xu, Hu Xu, Yuan Zhou

**Affiliations:** ^1^Interventional Therapy Department, Nantong Tumor Hospital, Nantong, Jiangsu Province, China; ^2^Department of Oncology, Nantong Tumor Hospital, Nantong, Jiangsu Province, China; ^3^Hepatology Department, Nantong Tumor Hospital, Nantong, Jiangsu Province, China

## Abstract

**Background:**

A number of researches focused on the study of tumors have concluded that the expression level of lncRNA *NKILA* was decreased in different tumors. This is an indication that *NKILA* might influence the start and growth of a cancer. In addition, studies have fatalities and worsening health of cancer patients is associated with a reduced level of *NKILA*.

**Results:**

The results are the collective screening of nine total studies which included 937 cancer patients. The prognosis of the meta-analysis indicated that cancer patients with a higher expression of *NKILA* had an overall longer survival (OS) (HR = 0.808, 95% CI: 0.736, 0.887); with regard to the clinical prognosis, the results indicated that reduced *NKILA* was associated with advanced clinical stage (OR = 0.313, 95% CI: 0.225, 0.434), poor histological grades (OR = 0.833, 95% CI: 0.508, 1.367), positive lymph node metastasis (OR = 0.253, 95% CI: 0.144, 0.444), and additional tumor invasion depth (OR = 0.326, 95% CI: 0.234, 0.454).

**Materials and Methods:**

Related research conducted was accessed by searching in PubMed and Web of Science with the keywords. The accessed material was till the 25^th^ of February, 2020. The present quantitative meta-analysis was done using Stata SE12.0. The aim of the meta-analysis was to investigate the relationship between *NKILA* expression level and clinical prognosis.

**Conclusions:**

In the result of this meta-analysis, decreased *NKILA* expression is typical of different kinds of cancer. Moreover, it can perform as a predictive element of prognosis in varied kinds of cancer. Nonetheless, till now, it is deemed essential to carry out larger-size as well as better designed research works for the confirmation of our findings.

## 1. Introduction

Long noncoding RNAs (lncRNAs) are involved in a number of physiological and pathological processes. They belong to a large class of RNAs with a capacity for non-protein-coding. In other words, they are defined as transcripts with lengths that are greater than two hundred bases [1]. A majority of lncRNAs exhibit similar characteristics when exerting biological functions. They achieve this by regulating various aspects of gene expression [2, 3]. Even though accurate mechanisms by which lncRNAs function has not been explained to date, research has indicated that a majority of the lncRNAs exhibit similar characteristics which are important such as proliferation [4], apoptosis [5], metastasis [6], metabolism [7], senescence [8], and drug resistance [9]. With regard to their functioning mechanism, a model which has been widely proposed is that lncRNAs may be functioning through discrete modules that decoy, guide, or scaffold other regulatory proteins [1].

Some have the potential to result in prooncogene. Contrastingly, other studies have also introduced low expression with regard to the performance of functions that suppress tumors [10–13]. The present study emphasises on the lncRNA, which is known as *NKILA*, a NF-*κ*B-induced lncRNA; in comparison to others, it is a novel form of lncRNA that maps to chromosome 20q13 [14, 15]. Studies have documented that it has useful functionality in suppressing the growth of cancer genes in a variety of cancers, such as esophageal squamous cell carcinoma [16], laryngeal cancer [17], nasopharyngeal carcinoma [18], rectal cancer [19], tongue squamous cell carcinoma [20], and colorectal cancer [21].

*NKILA*, whose expression is induced by NF-*κ*B, attaches to NF-*κ*B/I*κ*B and obscures phosphorylation motifs of I*κ*B; as a result, the actions of IKK-mediated I*κ*B phosphorylation and NF-*κ*B are suppressed. Eventually, it assumes the role of suppressing tumors in various cancers, like esophageal squamous cell carcinoma [16], laryngeal cancer [17], nasopharyngeal carcinoma [18], and hepatocellular carcinoma [22]. Moreover, NKILA-mediated NF-*κ*B activation can be regarded as a therapeutic molecule since it inhibits EMT and suppresses the advancement of cancer; this is evident in breast cancer [23], hepatocellular carcinoma [24], osteosarcoma [25], and non-small-cell lung cancer [26]. Meanwhile, it was demonstrated by Liu and Shi that the role of *NKILA* in the deterioration of NSCLC is dependent on the IL-11/STAT3 signalling [27]. In the meantime, it was also demonstrated that lncRNA was related to the immune evasion of tumors. In addition, an investigation by Huang et al. revealed that *NKILA* preferentially sensitizes antitumor T cells to cell death following activation by tumor antigens [28]. The study demonstrated that reducing the activity of *NKILA* intensifies the infiltration of CTL which results in the inhabitation in the expansion and development of the tumor. As a result, it indicates a potential methodology for improving the effectiveness of T cells in adoptive T cell therapy in the treatment of cancers [28].

In order to validate the medical usefulness and effectivity as a biomarker or therapeutic target, it is vital to study the relationship of *NKILA* expression level to any pathological characteristics. The work done in the present study is aimed at fulfilling an organized review and performing a quantitative meta-analysis to investigate the relationship between the expression level of *NKILA* and various forms of cancers occurring in humans.

## 2. Results

### 2.1. Data Selection and Characteristics

**Figure 1:**
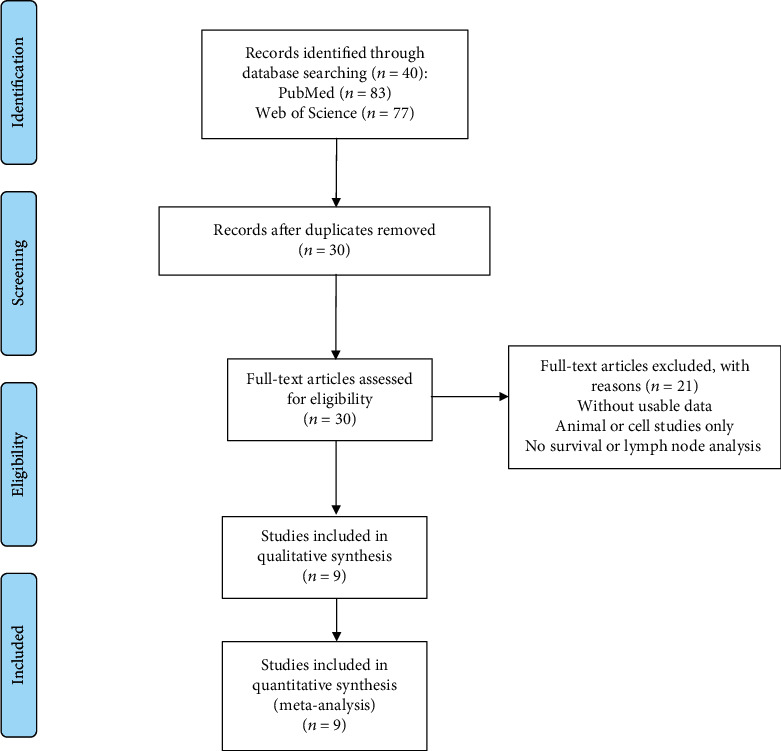
Flowchart used for selecting studies for inclusion.

The present study analyses the collective results of nine researches involving 937 cancer patients in total. All participants were accommodated according to the criteria for being suitable participants for the study. All the research work originated from China. Two studies emphasised on hepatocellular carcinoma, one study was related to colorectal cancer, whilst one other emphasised on rectal cancer, whilst another study was associated with osteosarcoma. Apart from these, the other studies, respectively, concentrated on laryngeal cancer, non-small-cell lung cancer, tongue squamous cell carcinoma, esophageal squamous cell carcinoma, and nasopharyngeal carcinoma. Quantitative reverse transcription polymerase chain reaction (RT-qPCR) was applied to detect *NKILA*. Based on these results, the patients were grouped as having either high *NKILA* expression or low *NKILA* expression.  Table 1 lists the summarized characteristics of the studies whilst Figure 1 depicts the flow chart of the research study and selection.

### 2.2. Association between *NKILA* Expression and Pathological Features

#### 2.2.1. Clinical Stage

**Figure 2:**
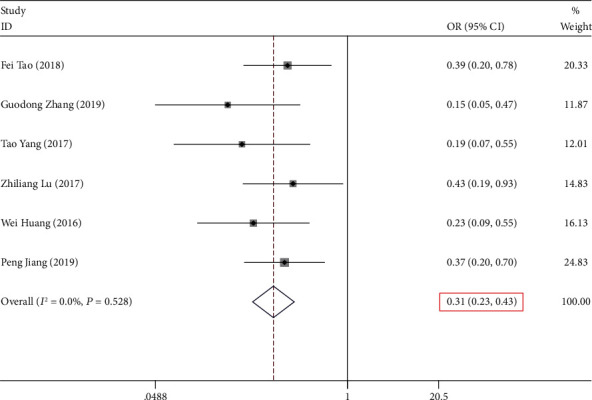
Forest plot for the association between *NKILA* expression and clinical stage in human cancers.

A total of 7 researches offered reports in the relationship between lncRNA *NKILA* expression and clinical stage (III/IV versus I/II). The heterogeneity of the considered researches was found to be not statistically significant (*P* > 0.05, *I*^2^ = 0.00%). As a result, the fixed-effects model was utilized to perform the calculation of the pooled OR, combined with its 95% CI, which amounted to be significantly different (OR = 0.313, 95% CI (0.225, 0.4234), and *P* ≤ 0.001) (Figure 2, Table 2). This result implied that a low expression level of *NKILA* was indeed related with advanced clinical stage.

#### 2.2.2. Histological Grade

**Figure 3:**
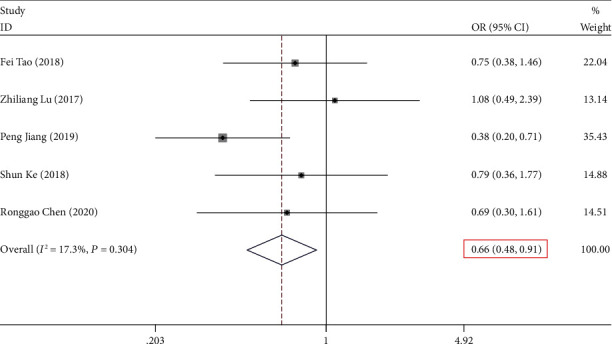
Forest plot for the association between *NKILA* expression and histological grade in human cancers.

A collection of five studies reported the relationship between expression and histological grade. It was determined that the heterogeneity in the considered studies was not statistically significant (*P* > 0.05, *I*^2^ = 17.30%). As a result, the random-effects model was utilized to perform the calculation of the pooled OR, combined with its 95% CI, which was significantly different (OR = 0.661, 95% CI (0.478, 0.914), and *P* ≤ 0.05) (Figure 3, Table 2). This indicates that expression was significantly distinguished with the histological grade.

#### 2.2.3. Lymph Node Metastasis

**Figure 4:**
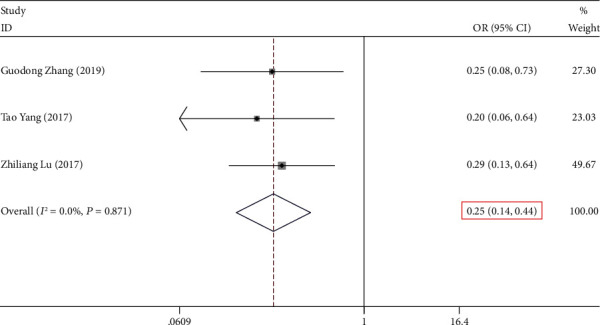
Forest plot for the association between *NKILA* expression and lymph node metastasis in human cancers.

A collection of three studies presented findings on the relationship between *NKILA* expression and lymph node metastasis. The heterogeneity in the collective results did not appear as statistically significant (*P* > 0.05, *I*^2^ = 0.00%). Consequently, fixed-effects model was utilized to perform the calculation of the pooled OR, combined with its 95% CI, which was significantly different (OR = 0.253, 95% CI (0.144, 0.444), and *P* ≤ 0.001) (Figure 4, Table 2). The results imply that the cohort of low *NKILA* expression level was susceptible to a heightened risk of lymph node metastasis in comparison with that of high *NKILA* expression level.

#### 2.2.4. Tumor Invasion Depth (T)

**Figure 5:**
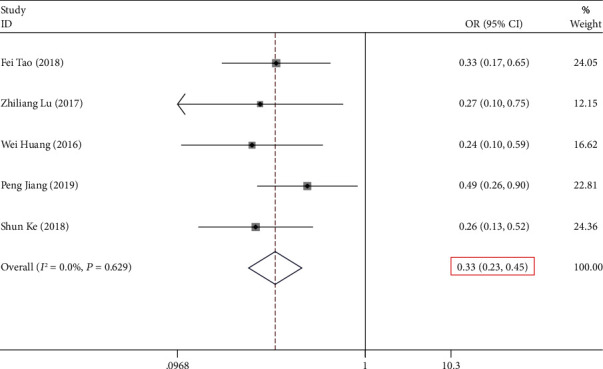
Forest plot for the association between *NKILA* expression and tumor invasion depth in human cancers.

The collective results of six studies reported the relationship between *NKILA* expression and tumor invasion depth. The heterogeneity analysis in the collective results did not appear to be statistically significant (*P* > 0.05, *I*^2^ = 0.00%). As a result, the fixed-effects model was utilized to perform the calculation of the pooled OR, combined with its 95% CI; it was determined to be significantly different (OR = 0.326, 95% CI (0.234, 0.454), and *P* ≤ 0.001) (Figure 5, Table 2). Based on this result, in comparison with that of high *NKILA* expression level, the cohort of low NKILA expression level possessed a heightened risk of deep tumor attack (T3 stage or above).

### 2.3. Association between *NKILA* Expression and Survival in Different Types of Cancers

**Figure 6:**
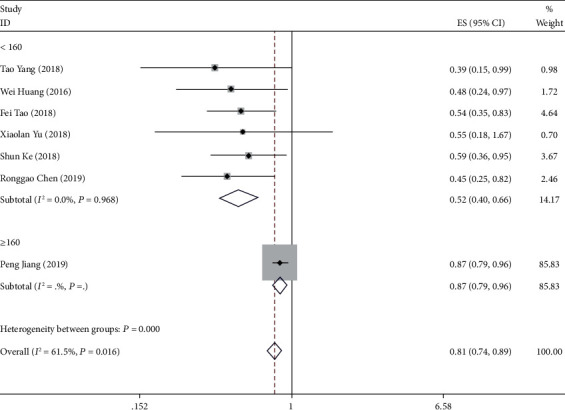
Meta-analysis for the pooled HRs of OS in patients with various cancers.

The collective results of seven studies involving 767 patients were used to evaluate the effect of *NKILA* overexpression on OS in various cancers. Overall survival characteristics of the included studies are shown in Table 3.The results suggested that elevated low NKILA expression projected a deteriorated result for OS with regard to six forms of cancer (pooled HR = 0.808, 95% CI 0.736, 0.887) with heterogeneity (*I*^2^ = 61.50%, *P* ≤ 0.05). Moreover, according to the number of participants in the study, a subgroup analysis was also performed. Evidently, a strong relationship between *NKILA* low expression and poor OS was observed in the studies associated with a participant number of less than 160 (pooled HR = 0.517, 95% CI 0.404, 0.661) (Figure 6). In comparison with the group of high *NKILA* expression, the group of low *NKILA* expression exhibited a substantial statistical reduction in OS whilst exhibiting a correlation with the clinically adverse outcomes.

#### 2.3.1. Assessment of Publication Bias

In order to assess the publication partiality in the current study, a performance of Begg's funnel plot was completed. Following its implementation, there was no notable evidence observed in the publication partiality during the analysis of the clinical stage (*P* = 0.017), histological grade (*P* = 0.183), lymph node metastasis (*P* = 0.234), and also the tumor invasion depth (*P* = 0.221).

## 3. Conclusion

In the result of this meta-analysis, decreased *NKILA* expression is typical of different kinds of cancer. Moreover, it can perform as a predictive element of prognosis in varied kinds of cancer. Nonetheless, till now, it is deemed essential to carry out larger-size as well as better designed research works for the confirmation of our findings.

## 4. Discussion

Over the recent years, the increased regulation of lncRNAs is performed in various cancers occurring in humans. Apart from its relation with the progression of the disease, lncRNAs such as lncRNA *HOTAIR* [29] are effective in serving as molecular scaffolds, delivering particular regulatory proteins within proximity of each other. Thus, it is able to function as a distinctly complex structure. It will then have a vital role in the development and progression of a tumor. By regulating the transcription of *CDKN1A* epigenetically in the nucleus, lncRNA SNHG1 [2] is likely to promote malignancy of cholangiocarcinoma. Thus, it facilitates the survival and metastasis of the cells related to cholangiocarcinoma. Generally, these expressed lncRNAs and are expected to occur as molecular biomarkers. They are useful in developing therapeutic methods which would be effective in the diagnosis and treatment as well as prognosis in various cancers occurring in humans.

The aim of the present meta-analysis was to investigate the relationship between *NKILA* expression level and pathological attributes in human cancers. A total of 937 patients studied under nine distinct researches were implicated in the end. Based on the algorithmic meta-analysis, the results obtained from the present study indicate that cancer patients with a low level of *NKILA* expression have shorter OS. In addition, with regard to the clinical prognosis, the results indicated that the group associated with low *NKILA* expression level had a heightened risk of advanced clinical stage, poor histological grades, positive lymph node metastasis, and extended tumor invasion depth.

However, the present study had a few considerable limitations which are listed below:

1. There was a geographical limitation since all patients involved in the study were from China. This excludes patients from different nationalities
2. The number of participating patients was limited since only a small number was considered for each research. In addition, only a few types of cancer were investigated
3. There was no observed consensus on the cutoff approximated which is needed to distinguish between high and low *NKILA* expression levels
4. There was a lack of cohort research which satisfied the inclusion criteria. A combination of an increased sample-size and improved quality in research will further guarantee the results obtained in this study

In summary, a higher expression level of lncRNA *NKILA* exhibited a close relationship with poorly distinguished grade, deep tumor invasion, and lymph node metastasis. This suggested that it had the capacity to serve as a biomarker of poor prognosis for cancer patients.

## 5. Materials and Methods

### 5.1. Literature Search Strategies

Two scholars independently identified the related literature. This was achieved using PubMed and Web of Science. The goal was to source studies related to investigating the relationship between *NKILA* expression level and pathological characteristics in cancer patients. A mix of keywords (“*NKILA*”, “cancer or carcinoma or carcinoma or tumor or neoplasm”) was used in the literature search strategy. This was combined with the detection of relevant research work that supplemented the obtained literature.

### 5.2. Inclusion and Exclusion Criteria

The literature related to the present study was required to be in accordance with the inclusion criteria listed below:

1. Stated expression levels of *NKILA*, as figured out with the help of RT-qPCR
2. Provided the decision
3. Classified cancer patients according to two groups, high and low expression groups with the use of detailed criteria for *NKILA* expression levels
4. Reported findings with regard to the clinicopathological attributes of the patients; at least one of the following pathological attributes should have been reported: histological grade, tumor invasion depth, lymph node metastasis, clinical stage, and distant metastasis
5. It should be a case-control or cohort study

If the sourced literature for the present study had at least one of the features of the exclusion criteria, it was deemed to be unfit for the purpose of this study:

1. Researches that were recurring or ones that used patients stated in prior studies
2. Failure to adequately account for the data
3. Utilized nonhuman species in the experiments
4. Reflections, letters, unpublished data, and commentaries
5. Reports compiled in languages that were not English

The quality of the research was evaluated by two scholars; they studied the title, abstract, and the overall content of each report that referred to the inclusion as well as exclusion criteria.

### 5.3. Literature Screening and Data Extraction

The data was independently collected by two investigators (Siyuan Tian and Yang Yu). All instances of collected data were in accordance with both the inclusion and exclusion criteria. Any differences were resolved through consensus or discussion with a third investigator (Honghua Huang) before the commencement of analysing the collected data. Data derivation of literature was as follows: first author, publication year, country of origin, kind of cancer, total number of patients, number of patients assigned to both high and low *NKILA* expression groups, the identification approach, and the cutoff approximations for *NKILA* expression levels.

### 5.4. Quality Assessment

Quality assessment of the considered researches was performed using the Newcastle-Ottawa Scale standard; the scores were as follows: including selection (4 points), comparability (2 points), and outcome (3 points). The score was in the range of 0 to 9. Independent evaluation of each article was performed by 2 investigators (Siyuan Tian and Yang Yu); the differences were resolved through consensus or through discussion with a third investigator (Honghua Huang). Each entitled research work was scored in Table 1; a higher score was indicative of an enhanced methodological quality.

### 5.5. Statistical Analysis

Cochran's and chi-squared-based *Q* and *I*^2^ tests were utilized in order to determine the heterogeneity amongst the considered researches. The performance of the homogeneity test was completed for a significance level of *α* = 0.1. *P* values < 0.1 were considered significant, and *I*^2^ values > 50% represented the heterogeneity amongst the researches. The fixed-effects model was utilized for analysing the homogenous data. On the other hand, the random-effects model was utilized for analysing the heterogeneous data. Statistical analysis was carried out with the use of Stata SE 12.0 (Stata Corp LP, College Station, Texas, USA); these were also utilized for the assessment of publication partiality.

## Figures and Tables

**Table 1:** Clinical characteristics of the included studies.

Surname (year)	Country	Cancer type	Sample size	High	Low	Cutoff (high/low)	Detection method	Clinical features	Quality score	References
Fei Tao (2018)	China	Rectal cancer	152	76	76	Median	qRT-PCR	C; T; H	7	[19]
Guodong Zhang (2019)	China	Osteosarcoma	60	30	30	Median	qRT-PCR	C; L	6	[25]
Tao Yang (2017)	China	Laryngeal cancer	65	33	32	Median	qRT-PCR	C; L; H	7	[17]
Zhiliang Lu (2017)	China	NSCLC	110	55	55	Median	qRT-PCR	C; H; T; L	8	[26]
Wei Huang (2016)	China	TSCC	96	44	52	Mean	qRT-PCR	C; H; T	7	[20]
Peng Jiang (2019)	China	CRC	173	71	102	Mean	qRT-PCR	C; H; T	6	[21]
Shun Ke (2018)	China	ESCC	137	68	69	Median	qRT-PCR	C; H	8	[16]
Ronggao Chen (2020)	China	HCC	90	45	45	Median	qRT-PCR	H	7	[24]
Xiaolan Yu (2018)	China	HCC	54	27	27	Median	qRT-PCR	NA	7	[22]

NSCLC: non-small-cell lung cancer; TSCC: tongue squamous cell carcinoma; CRC: colorectal cancer; ESCC: esophageal squamous cell carcinoma; HCC: hepatocellular carcinoma; C: clinical stage; H: histological grade; L: lymph node metastasis; T: tumor invasion depth; NA: not available.

**Table 2:** Meta-analysis results for the association of overexpressed *NKILA* with clinicopathological parameters.

Clinicopathological parameters	Studies (*n*)	Numbers of patients	OR (95% CI)	*P* value	Heterogeneity		
					*I* ^2^	pH	Model
Clinical stage (III/IV vs. I/II)	6	652	0.313 (0.225, 0.434)	≤0.001	0.00%	>0.05	Fixed effects
Histological grade (poorly/others vs. well/moderately)	5	658	0.661 (0.478, 0.914)	≤0.05	17.30%	>0.05	Fixed effects
Lymph node metastasis (+ vs. -)	3	231	0.253 (0.144, 0.444)	≤0.001	0.00%	>0.05	Fixed effects
Tumor invasion depth (T3/T4 vs. T1/T2)	5	664	0.326 (0.234, 0.454)	≤0.001	0.00%	>0.05	Fixed effects

**Table 3:** Overall survival characteristics of the included studies.

Surname (year)	Country	Cancer type	Survival analysis	HR statistic	Hazard ratios (95% CI)	Follow-up months	Outcome
Tao Yang (2018)	China	Laryngeal cancer	Univariate Multivariate	Data in paper	0.389 (0.152, 0.993)	60	OS
Wei Huang (2016)	China	TSCC	Univariate Multivariate	Data in paper	0.476 (0.236, 0.975)	80	OS, DFS
Fei Tao (2018)	China	Rectal cancer	Univariate Multivariate	Data in paper	0.540 (0.350, 0.830)	96	OS
Peng Jiang (2019)	China	CRC	Univariate Multivariate	Data in paper	0.870 (0.787, 0.962)	72	OS, DFS
Xiaolan Yu (2018)	China	HCC	NA	Survival curves	0.550 (0.180, 1.670)	60	OS
Shun Ke (2018)	China	ESCC	Univariate Multivariate	Data in paper	0.590 (0.360, 0.950)	100	OS, DFS
Ronggao Chen (2019)	China	HCC	Univariate Multivariate	Data in paper	0.454 (0.251, 0.822)	NA	OS

TSCC: tongue squamous cell carcinoma; CRC: colorectal cancer; HCC: hepatocellular carcinoma; ESCC: esophageal squamous cell carcinoma; NPC: nasopharyngeal carcinoma; Univariate: univariate analysis; Multivariate: multivariate analysis; NA: not available; OS: overall survival; DFS: disease-free survival.

## References

[1] Liu B., Sun L., Liu Q. (2015). A cytoplasmic NF-*κ*B interacting long noncoding RNA blocks I*κ*B phosphorylation and suppresses breast cancer metastasis. *Cancer Cell*.

[2] Yu Y., Zhang M., Wang N. (2018). Epigenetic silencing of tumor suppressor gene CDKN1A by oncogenic long non-coding RNA SNHG1 in cholangiocarcinoma. *Cell Death & Disease*.

[3] Xu Y., Lian Y., Zhang Y. (2018). The long non-coding RNA PVT1 represses ANGPTL4 transcription through binding with EZH2 in trophoblast cell. *Journal of Cellular and Molecular Medicine*.

[4] Yang L., Lin C., Jin C. (2013). lncRNA-dependent mechanisms of androgen-receptor-regulated gene activation programs. *Nature*.

[5] Huarte M., Guttman M., Feldser D. (2010). A large intergenic noncoding RNA induced by p53 mediates global gene repression in the p53 response. *Cell*.

[6] Yuan J. H., Yang F., Wang F. (2014). A long noncoding RNA activated by TGF-*β* promotes the invasion-metastasis cascade in hepatocellular carcinoma. *Cancer Cell*.

[7] Yang F., Zhang H., Mei Y., Wu M. (2014). Reciprocal regulation of HIF-1*α* and lincRNA-p21 modulates the Warburg effect. *Molecular Cell*.

[8] Yap K. L., Li S., Muñoz-Cabello A. M. (2010). Molecular interplay of the noncoding RNA ANRIL and methylated histone H3 lysine 27 by polycomb CBX7 in transcriptional silencing of INK4a. *Molecular Cell*.

[9] Fan Y., Shen B., Tan M. (2014). Long non-coding RNA UCA1 increases chemoresistance of bladder cancer cells by regulating Wnt signaling. *The FEBS Journal*.

[10] Li Z., Yu X., Shen J. (2016). ANRIL: a pivotal tumor suppressor long non-coding RNA in human cancers. *Tumor Biology*.

[11] Sun M., Jin F. Y., Xia R. (2014). Decreased expression of long noncoding RNA GAS5 indicates a poor prognosis and promotes cell proliferation in gastric cancer. *BMC Cancer*.

[12] Gutschner T., Hammerle M., Eissmann M. (2013). The noncoding RNA MALAT1 is a critical regulator of the metastasis phenotype of lung cancer cells. *Cancer Research*.

[13] Zhang E. B., Kong R., Yin D. D. (2014). Long noncoding RNA ANRIL indicates a poor prognosis of gastric cancer and promotes tumor growth by epigenetically silencing of miR-99a/miR-449a. *Oncotarget*.

[14] Hung T., Wang Y., Lin M. F. (2011). Extensive and coordinated transcription of noncoding RNAs within cell-cycle promoters. *Nature Genetics*.

[15] Dalvai M., Mondesert O., Bugler B., Manenti S., Ducommun B., Dozier C. (2013). Doxorubicin promotes transcriptional upregulation of Cdc25B in cancer cells by releasing Sp1 from the promoter. *Oncogene*.

[16] Ke S., Li R. C., Meng F. K., Fang M. H. (2018). NKILA inhibits NF-*κ*B signaling and suppresses tumor metastasis. *Aging*.

[17] Yang T., Li S., Liu J., Yin D., Yang X., Tang Q. (2018). lncRNA-NKILA/NF-*κ*B feedback loop modulates laryngeal cancer cell proliferation, invasion, and radioresistance. *Cancer Medicine*.

[18] Zhang W., Guo Q., Liu G. (2019). NKILA represses nasopharyngeal carcinoma carcinogenesis and metastasis by NF-*κ*B pathway inhibition. *PLoS Genetics*.

[19] Tao F., Xu Y., Yang D. (2018). LncRNA NKILA correlates with the malignant status and serves as a tumor-suppressive role in rectal cancer. *Journal of Cellular Biochemistry*.

[20] Huang W., Cui X., Chen J. (2016). Long non-coding RNA NKILA inhibits migration and invasion of tongue squamous cell carcinoma cells via suppressing epithelial-mesenchymal transition. *Oncotarget*.

[21] Jiang P., Han X., Zheng Y., Sui J., Bi W. (2019). Long non-coding RNA NKILA serves as a biomarker in the early diagnosis and prognosis of patients with colorectal cancer. *Oncology Letters*.

[22] Yu X., Tang W., Yang Y. (2018). Long noncoding RNA NKILA enhances the anti-cancer effects of baicalein in hepatocellular carcinoma via the regulation of NF-*κ*B signaling. *Chemico-Biological Interactions*.

[23] Wu W., Chen F., Cui X. (2018). LncRNA NKILA suppresses TGF-*β*-induced epithelial-mesenchymal transition by blocking NF-*κ*B signaling in breast cancer. *International Journal of Cancer*.

[24] Chen R., Cheng Q., Owusu-Ansah K. G. (2020). NKILA, a prognostic indicator, inhibits tumor metastasis by suppressing NF-*κ*B/Slug mediated epithelial-mesenchymal transition in hepatocellular carcinoma. *International Journal of Biological Sciences*.

[25] Zhang G. D., Li Y., Liao G. J., Qiu H. W. (2019). LncRNA NKILA inhibits invasion and migration of osteosarcoma cells via NF-*κ*B/Snail signaling pathway. *European Review for Medical and Pharmacological Sciences*.

[26] Lu Z., Li Y., Wang J. (2017). Long non-coding RNA NKILA inhibits migration and invasion of non-small cell lung cancer via NF-*κ*B/Snail pathway. *Journal of Experimental & Clinical Cancer Research*.

[27] Liu D., Shi X. (2019). Long non-coding RNA NKILA inhibits proliferation and migration of lung cancer via IL-11/STAT3 signaling. *International Journal of Clinical and Experimental Pathology*.

[28] Huang D., Chen J., Yang L. (2018). NKILA lncRNA promotes tumor immune evasion by sensitizing T cells to activation-induced cell death. *Nature Immunology*.

[29] Tsai M. C., Manor O., Wan Y. (2010). Long noncoding RNA as modular scaffold of histone modification complexes. *Science*.

